# Complete mitochondrial genome of Min pig in nucleus herd

**DOI:** 10.1080/23802359.2020.1791748

**Published:** 2020-09-16

**Authors:** Lijuan Wang, Li Wang, Jianming Liu, Shengwei Di

**Affiliations:** a College of animal science and technology, Northeast Agricultural University, Harbin, China; b College of Agricultural, Eastern Liaoning University, Dandong, China; c Yili Animal Science Research Institute, YiNing, China

**Keywords:** Min pig, mitochondrial genome, nucleus herd, species conservation

## Abstract

In this study, the complete mitochondrial genome of Min pig (*Sus scrofa*) in nucleus herd was sequenced in order to develop the mitogenome data for genus *Sus*. The complete mitogenome sequence is 16,719 bp in length, containing a D-loop region, 13 protein-coding genes, 2 ribosomal RNA, and 22 transfer RNA genes. The new sequenced complete mitogenome of Min pig in nucleus herd will provide useful information for application in conservation genetics and evolution for this Near Threatened pig genomes.

Min pig (*Sus scrofa*), a local breed in Northeastern China, has been characterized as prolific (14.35 piglets per birth) and a source of good meat, and has a strong resistance to poor feed quality and cold climate (Wang et al. 2002; Song et al. 2018). Furthermore, its good maternal temperament is another outstanding characteristic of the Min pig breed (Wang et al. 2002). However, the number of Pure-Breed Min pig decreased sharply year by year because of the introduction of foreign breeds with a high growth rate and a high lean meat percentage. In addition, irrational use of Min pig germplasm resources and breed degraded made this excellent breed tend to become extinct and the number less than 500 in the elite reservation farm Min pig. Mitochondrial DNA was a useful marker to trace back the origin of livestock species and had been widely used to reconstruct domestication patterns (Jin et al. 2012). To research the genetic resource of Min pig, we reported the mitochondrial genome of Min pig in nucleus herd.

The ear tissue sample of Min pig used for this study was collected from the Lanxi pig breeding farm (elite reservation farm of Min pig), Suihua, Heilongjiang Province, China (126.28°E, 46.24°N), in 2016. DNA was extracted from ear tissue and was preserved Northeast Agricultural University and designated 498-1. The mitogenomes were amplified using 14 primers referenced from the *S. scrofa* breed Meishan (KM998967.1), and PCR products were determined by the method of Sanger sequencing. The mitogenome of Min pig is 16,719 bp in length and was deposited in the GenBank under accession number MT653633. The overall nucleotide composition was 34.6% A, 25.8% T, 26.2% C, and 13.4% G, with a total A and T content of 60.4%. It has 37 mitochondrial genes, including 2 rRNA (*12S rRNA* and *16S rRNA*) genes, 13 protein-coding genes (PCGs), 22 tRNA genes, and a noncoding region.

To validate the phylogenetic position of Min pig, a maximum-likelihood phylogenetic tree of 20 *S. scrofa* breeds was constructed based on the complete mitochondrial DNA sequence. As shown in the phylogenetic tree (Figure 1), Min pig is closely related to Dapulian pig and Laiwu pig, which is consistent with the traditional regional classification. The three foreign breeds of Hampshire, Pietrain, and Duroc are far apart from Chinese cluster distribution. This can be explained by the pigs that are independently domesticated in various regions of the world (Yang et al. 2003; Jin et al. 2012).

**Figure 1.**
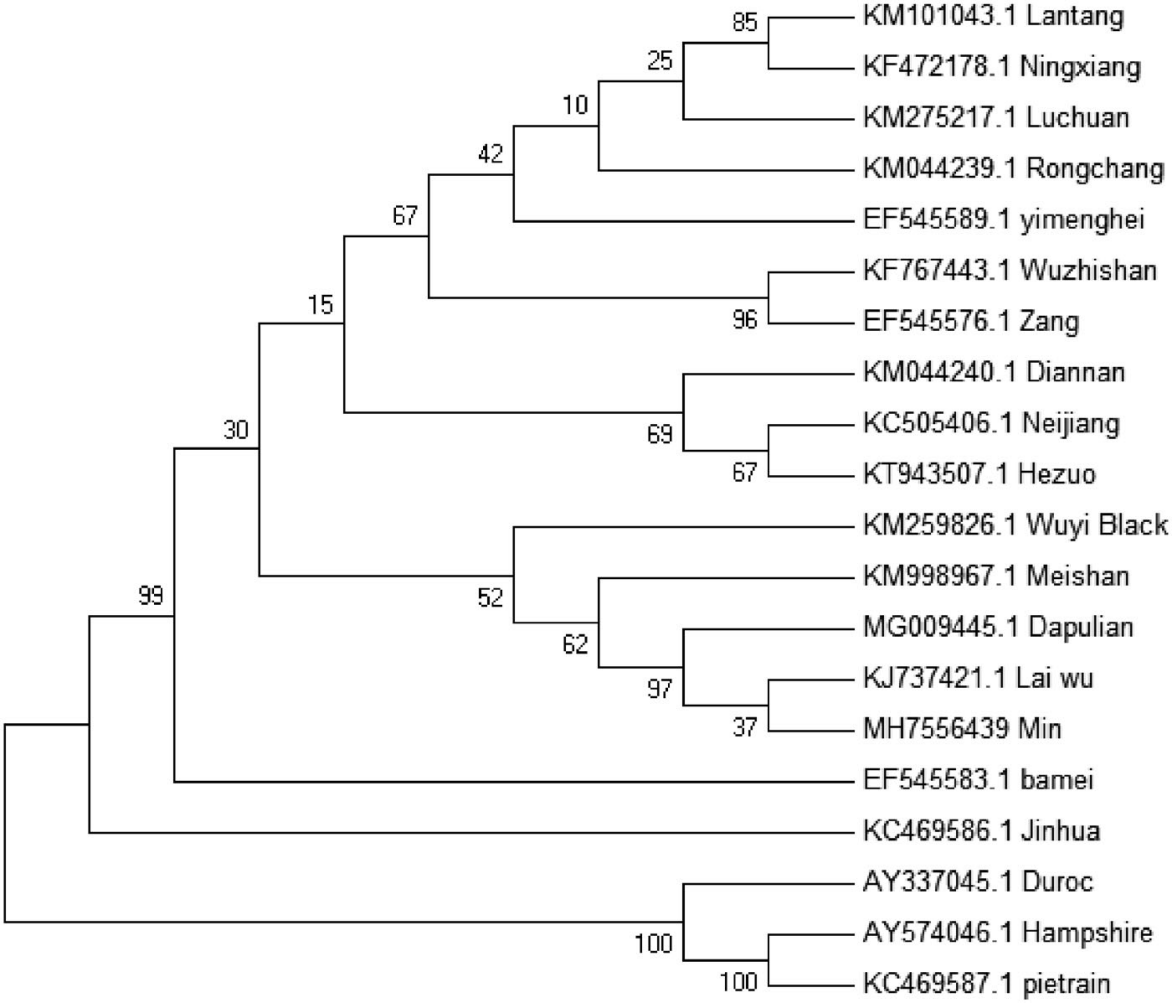
Phylogenetic relationships (maximum-likelihood) among the species of the *Sus scrofa* based on the mitochondrial genome nucleotide sequence. Numbers beside the nodes are percentages of 1000 bootstrap values. Alphanumeric terms indicate the GenBank accession numbers.

## Data Availability

The data that support the findings of this study are openly available in ‘NCBI’ at https://www.ncbi.nlm.nih.gov/, reference number MT653633.
